# Prevalence of Learned Grapheme-Color Pairings in a Large Online Sample of Synesthetes

**DOI:** 10.1371/journal.pone.0118996

**Published:** 2015-03-04

**Authors:** Nathan Witthoft, Jonathan Winawer, David M. Eagleman

**Affiliations:** 1 Department of Psychology, Stanford University, Stanford, California, United States of America; 2 Department of Psychology and Center for Neural Science, New York University, New York, New York, United States of America; 3 Department of Neuroscience, Baylor College of Medicine, Houston, Texas, United States of America; University Zurich, SWITZERLAND

## Abstract

In this paper we estimate the minimum prevalence of grapheme-color synesthetes with letter-color matches learned from an external stimulus, by analyzing a large sample of English-speaking grapheme-color synesthetes. We find that at least 6% (400/6588 participants) of the total sample learned many of their matches from a widely available colored letter toy. Among those born in the decade after the toy began to be manufactured, the proportion of synesthetes with learned letter-color pairings approaches 15% for some 5-year periods. Among those born 5 years or more before it was manufactured, none have colors learned from the toy. Analysis of the letter-color matching data suggests the only difference between synesthetes with matches to the toy and those without is exposure to the stimulus. These data indicate learning of letter-color pairings from external contingencies can occur in a substantial fraction of synesthetes, and are consistent with the hypothesis that grapheme-color synesthesia is a kind of conditioned mental imagery.

## Introduction

When a grapheme-color (GC) synesthete reads this sentence or other black printed text, they report that they ‘see’ the letters as colored. For a given GC synesthete, the particular color associated with each letter appears to be highly consistent over time, and showing consistent letter-color matching has become a requirement for classifying a subject as a synesthete [1]. Researchers have speculated since the mid 19th century on the origins of the letter color pairings [2, 3, 4, 5] and recent work has focused on the way that letter-color correspondences may be shaped by generally available environmental influences such as color words, alphabetical order, frequency of the letter in the language, and similarities between the shape or sound of different letters [6, 7, 8, 9, 10].

In a very small number of cases (n = 11), we have shown the correspondences between letters and colors can be directly learned from environmental contingencies such as colored letters available during childhood [11, 12]. However, some researchers have reasonably questioned the relevance of learning external correspondences to understanding GC synesthesia [11] due to its apparent rarity [13,14]. Not only are there very few documented cases, but a study specifically designed to discover childhood influences in a fairly large sample of Australian synesthetes failed to find any definitive examples [6].

In this paper we examine the grapheme-color pairings of 6588 American synesthetes with the goal of setting a minimum prevalence of associative learning in shaping grapheme-color correspondences. A relatively high prevalence would have implications not only for understanding synesthesia, but also how synesthesia may relate to ordinary cognition. If letter-color pairings in GC synesthesia can be learned from experience, this would provide a link between GC synesthesia, observed in a relatively small fraction of the population, and much more widespread learned multisensory associations found in most people, such as the sound of words experienced while reading [15]. In this paper, we address two questions. First, what fraction of a large population of GC synesthetes learned their colors from experience with colored letters? Second, do these synesthetes share learning mechanisms with other GC synesthetes, or are they a distinct subgroup that is more pre-disposed to learn letter—color pairings from their environment?

## Methods

6588 synesthetes provided demographic information and performed tasks online at www.synesthete.org. Participants checked a box agreeing to the use of their data (excluding personal information) before beginning the tasks. The study and method of consent were both approved by the Baylor College of Medicine IRB (protocol number: H-20366). Participants were not actively recruited to the study, but instead discovered the Synesthesia Battery online, through a combination of searches, word of mouth, and citations to the battery in the literature. The battery includes two measures designed to verify participants’ synesthesia [1]. One measure is the consistency of letter-color matches, computed as the city-block distance in RGB space between different color matches for each letter. Each letter is matched 3 times, with the letters presented in random order. The distance is summed across the 3 matches per letter, and averaged across the 26 letters. The score expected from a uniform random selection of RGB values is 3. To be classified as a synesthete, a subject must have a mean distance of less than 1 [1]. In our population of 6588 synesthetes, all subjects had a distance measure of 1 or less, with a median distance of 0.676 (95% CI = .670–.682; all reported confidence intervals were obtained using bootstrapping). The second metric is accuracy in a speeded congruency test, in which participants are shown letters with a color that is the same or different from their previous matches. The median accuracy in the sample was 90.32% (95% CI = 90.28–90.63)

For our analyses, we converted each RGB triplet from our data sample to one of the 11 Berlin and Kay basic color labels (black, gray, white, red, orange, brown, yellow, green, blue, purple, pink) [16]. To create the conversion from RGB to labels, we down-sampled the 255 x 255 x 255 RGB space to 9x9x12. One of the authors (NW) assigned a color label to each of the 972 points in the RGB grid. RGB values from the participant data were then transformed to labels by interpolating to the geometrically nearest point in the down-sampled RGB space and taking the corresponding label. If participants did not generate a color match for a particular letter, that letter was assigned a ‘no match’ label.

## Results

**Fig 1:**
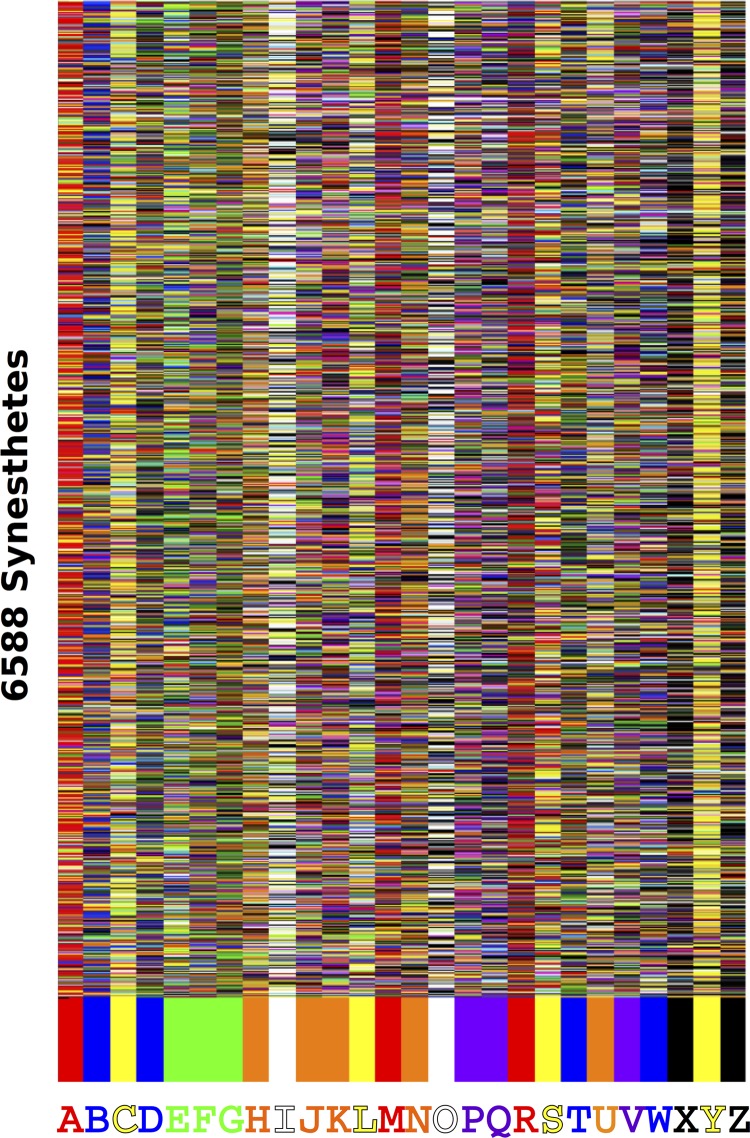
Grapheme-Color synesthesia in 6588 participants. The letter-color pairings across the whole population are shown with rows corresponding to participants and columns to letters. The colors along the bottom represent the most frequently chosen (modal) color label for each letter after interpolating from subject’s RGB coordinates to labels. Letters not assigned a color by participants are given a random color.

The entire data set is visualized in Fig. 1. Consistent with previous reports using English speakers [4, 6, 17], there are strong tendencies across the population for letters to be paired with particular colors (e.g., ‘Y’-> yellow). We will refer to the most frequently chosen color match for each letter hereafter as the ***modal choices*** or ***modal matching behavior*** for the population. A more thorough analysis of the structure of the data set as a whole and comparisons to other sets of letter-color matches, while interesting, is beyond the scope of the present paper.

**Fig 2:**
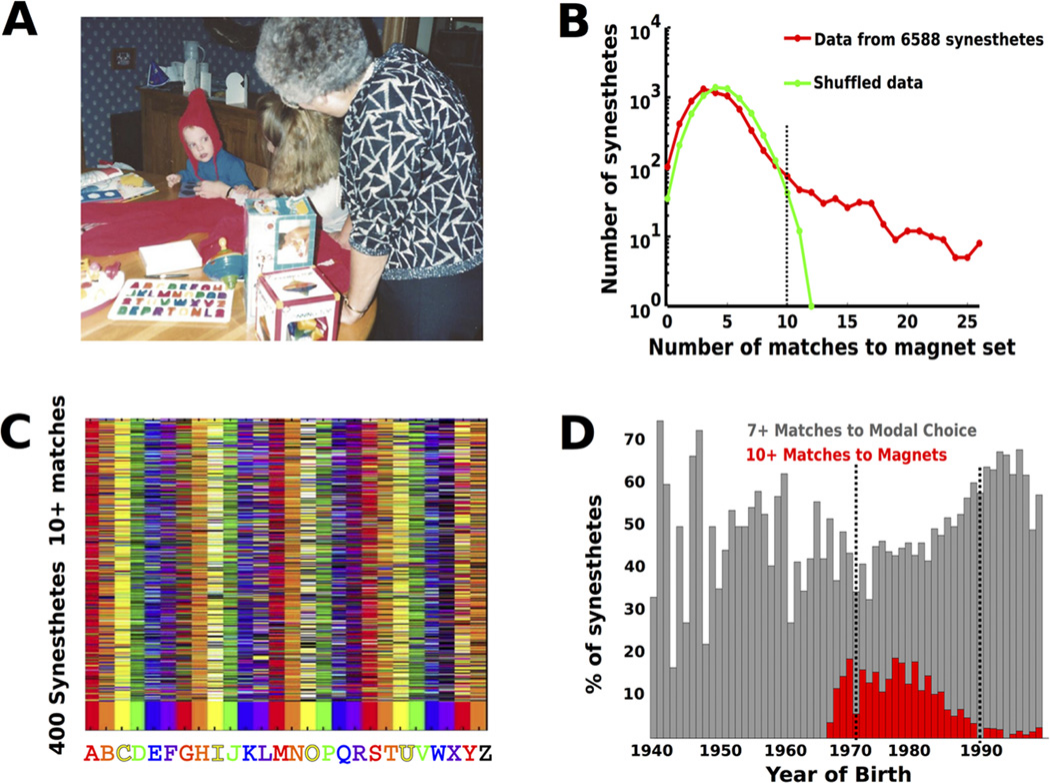
Prevalence of learned synesthesia in the sample. **A**. Photograph of the Fisher-Price letter set owned by a child who is now an adult synesthete with colors influenced by the toy. This participant was born in 1988 and has 25 matches to the colors in the toy. **B**. Histogram of the number of letter matches to the colors in the toy. The red curve shows the matches found in the empirical data while the green curve is derived from the shuffled data. In the shuffled distribution, the number of matches to the toy rarely exceeds 10 of 26 (black dashed line). In the empirical distribution, it can be as high as 26 of 26. **C**. Same as Fig. 1, except it shows only the 400 synesthetes with 10 or more letters matching the toy (those to the right of the black dashed line in panel B). **D**. Prevalence of magnet and modal matching over time. Red bars show proportion of participants with 10 or more matches to the magnet set as a function of year born. Grey bars show participants with 7 or more matches to modal choices. For participants born between 1970 and 1985, the prevalence of synesthesia apparently learned from the Fisher-Price set can exceed 15%.

What proportion of synesthetes in this data set learned their colors from some external stimulus? While we cannot know all possible stimuli that participants may have learned colors from, we can set a conservative baseline by looking for participants with matches corresponding to the colored letter magnet set we previously reported (Fig. 2A; [11, 12]). To find candidate participants, we first created a letter-color template by making a vector of color labels corresponding to the colors of the toy pictured in 2A. Then, for each subject in the database we computed the number of matches between their data and the template (Fig. 2B red line), with the idea that participants who learned their colors from this magnet set would have more matches than expected.

To define how many matches to the magnet set we would have expected to find in our participants by chance, we shuffled the 6588 color-letter pairings for each letter separately, thus preserving the non-uniform distribution of matches for each letter across colors. From the shuffled data, we created a null distribution composed of the number of matches each ‘participant’ in the shuffled data had to the template (Fig. 2B green line). Comparing our empirical and shuffled distributions shows that 400 participants (~6%, Fig. 2C) have 10 or more matches to the magnet set in the empirical data. The probability of 10 or more matches to the magnet set for a ‘participant’ in the shuffled data was less than 0.009% across 5000 shuffles (Fig. 2B dashed line). For brevity, we will refer to this group of 400 participants as the **magnet synesthetes**, though we stress that this is the source of the letter-color pairings rather than the cause of their synesthesia [11, 12].

Our randomization procedure controls for the general fact that the distribution of colors is different for each letter, and the particular issue that the modal choice and the magnet set are the same for some letters (e.g., A->red). This is because the colors are shuffled separately for each letter. Hence, just as many shuffled participants have red ‘A’s as actual participants, but for the shuffled participants, the probability that an A is red is independent of the probability that B is blue, and so on. Our comparison finds participants where the matches to the magnet set are too numerous to be accounted for by just the overall color matching tendencies for each letter across the population as a whole. We also note that our choice of null distribution is the most conservative with respect to our estimate of the number of magnet synesthetes. Other null distributions (such as assuming random uniform matches, or shuffling the data within each subject rather than within each letter) produce much higher estimates.

**Fig 3:**
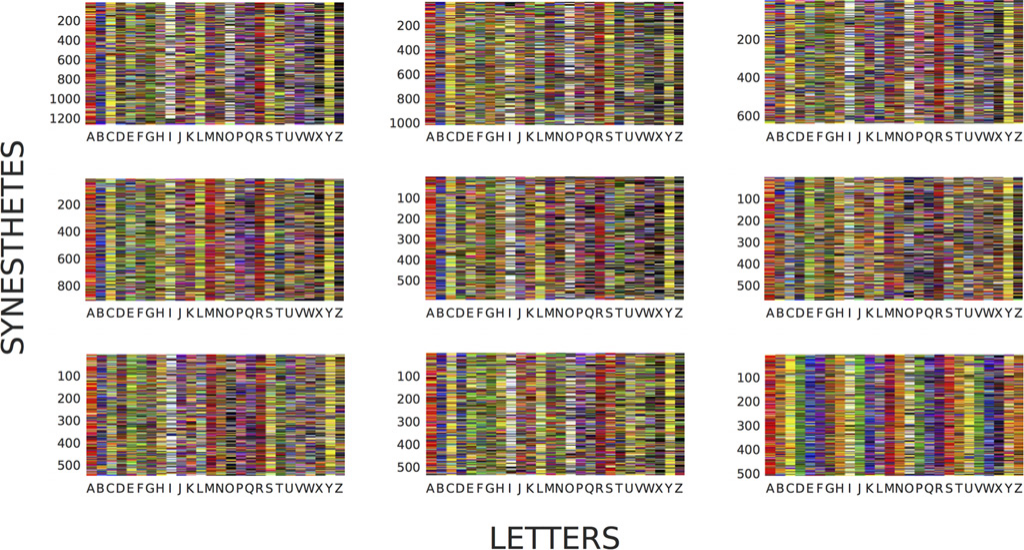
Clustering analysis. 9 clusters obtained by applying k-medoids algorithm to participants’ letter-color matching data (k = 9). A large cluster (n = 500) with data clearly resembling the magnet set can be seen at bottom right.

Are there other groups of participants with highly similar matches in our data that might be traced to some external source that we don’t know about? To answer this question, we explored the data using k-medoids clustering as implemented in MATLAB 2014b / version 8.4 (Mathworks Inc., Natick Massachusetts). Each participant was represented as a 26 element vector where each entry was a number from 1 to 12, with each number corresponding to one of the 11 color categories or no match (letters for which participants did not generate a match). For each pair of participants we defined the distance between the two participants as the number of letters for which they give different matches (Hamming distance). Fig. 3 shows the clusters obtained for k = 9 in one representative solution from the algorithm. There is one cluster of 500 synesthetes with matches clearly resembling the magnet set (Fig. 3, lower right). The other 8 groups are very similar to one another and for the most part reflect variations in the modal trends in the data. Over a thousand repetitions of k-medoids we found that 88.5% of the resulting clusters contained fewer than 40 of the 400 magnet synesthetes while 10.7% of the clusters contained more than 320 of the magnet synesthetes. This result is consistent with our previous analysis and suggests that no other large groups of similar consistency and size are present in this data set.

This particular toy was only produced between 1971 and 1990 [12]. Therefore, as an additional control we examined the birthdates of the 400 participants with more matches to the magnet set than expected given our null distribution. 6232 GC synesthetes in the dataset reported their birthdate, of whom 388 (~5.9%) had 10 or more matches to the magnets. The 388 participants were all born in a time frame where the set existed during their childhood (1967 or later), and our data show that the average prevalence of this type of learning for some 5-year periods approaches 15% (e.g., 1975–1980, Fig. 2D red bars). 3618 of the participants were born while the toy was being manufactured (1971–1990), and of these 329 (9.1%) were magnet synesthetes. For comparison we also examined the proportion of participants with more than 7 matches to the modal set of letter-color pairs as a function of birthdate (Fig. 2D grey bars). Synesthetes of this kind average 48.5% of the sample for the years 1940–2000 (only 14 participants gave birthdates earlier than 1940). Whatever drives the modal matching behavior in the data set has been continuously present, while the proportion of magnet synesthetes rises and falls with availability of the magnet set.

**Fig 4:**
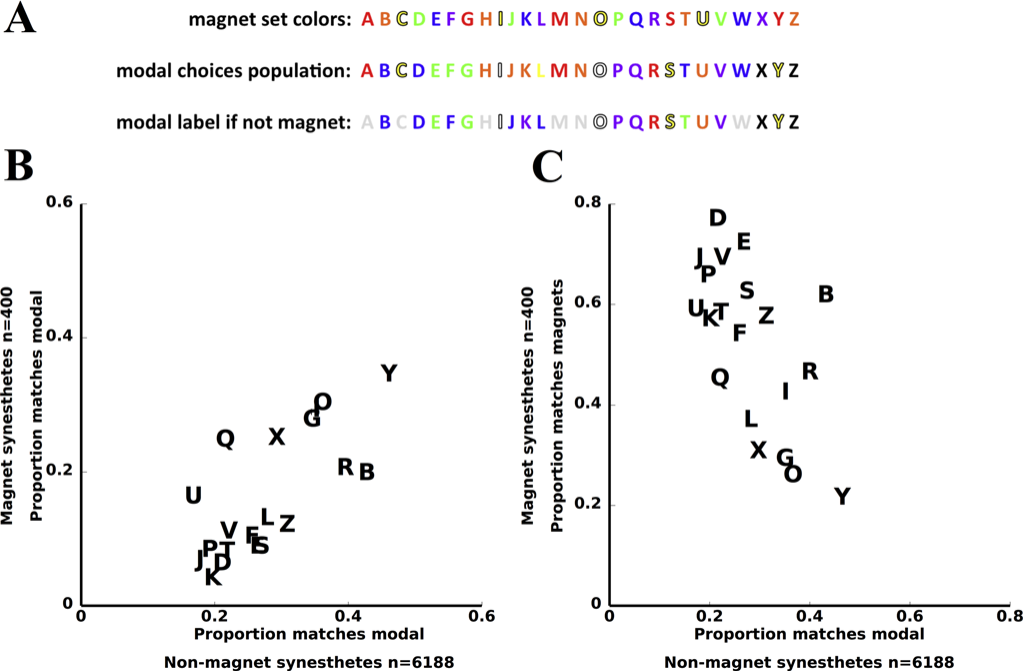
Synesthetes with learned matches are subject to the same influences as the larger population. **A**. The colors in the toy (upper row), the modal color choice for each letter from the 6188 synesthetes (middle row), and the most commonly assigned color for each letter for the 400 synesthetes from Fig. 2C, when the choice does not match the toy (bottom row). Letters for which the magnet set color is the same as the modal color for all synesthetes are indicated in gray. When the 400 synesthetes have letter color matches that don’t match the toy, they generally follow the same pattern as the rest of the population (15/20 letters the same). **B**. Relationship between the probability that a letter is matched to the modal choice in the 400 synesthetes from Fig. 2C and the other 6188 synesthetes. The strong positive correlation indicates that the two populations are subject to similar influences. **C**. Correlation between the probability that a latter matches the magnet set in the 400 synesthetes and the probability a letter matches the modal choice in the other 6158 synesthetes. The negative correlation signals competition between the two sources of influence in the magnet synesthetes, the toy versus general cultural/linguistic tendencies. Analyses in this section are limited to the subset of 20 letters which are not same for the magnet set and modal choice (excludes A, C, H, M, N, and W; grayed out in the bottom row of panel A).

Our data argue against the possibility that synesthetes who appear to have learned their grapheme-color pairings from some external source differ from the rest of this population of synesthetes. For example, when the 400 magnet-imprinted synesthetes have a letter-color pair that does not match the toy, the chosen color most often matches the modal choice of the other 6100+ synesthetes shown in Fig. 1 (Fig. 4A). In fact, the probability of making the modal choice is highly positively correlated across letters for the two groups (r = 0.72, p<0.0005, Fig. 4B). This suggests that whatever influences drive the modal matching behavior (e.g., the tendency to make B blue) also influences the letter-color associations of the magnet synesthetes. Moreover, the probability that a letter is matched to the magnet set in the 400 synesthetes is inversely correlated with how strong a tendency there is in the general population for a letter to be associated with a particular color, suggesting that whatever drives the color matching behavior in the larger population competes with the magnet set (r = -0.61, p<0.005, Fig. 4C). For example, letters such as Y and G, which are most frequently matched to yellow and green in the general population, are also the most frequently matched to yellow and green in participants with matches that largely follow the toy (where both letters are red).

## Discussion

That close to 1 in 6 GC synesthetes in a 10 year period appear to have learned their letter-color pairs from the same external source is not only surprising, but should be regarded as a *minimum bound*, as it reflects only one source of external influence that was presumably not available to the entire population of developing synesthetes. Other sets of alphabet colors may have been equally influential but less widely available, and therefore difficult to trace, such as a classroom poster, a parent’s mural, or letter toys by other manufacturers. Moreover, letter-color pairings might come from a mixture of external sources, which would be particularly difficult to track. Our estimate also neglects learning letter-color pairings from other sources of cultural information, such as obvious strong linguistic cues like the first letter of color words (see Fig. 1, b->blue, y->yellow, etc.) that show in the widespread and enduring influence that can be seen in Figs. 1 and 2D.

However, some might object that finding such a large proportion is entirely dependent on the fact that so many of these letter sets were made and sold in the U.S. We agree. In fact, the ubiquity of the letter set may be the main source of difference between our result and the null result in Rich et al 2005 [6], which used Australian synesthetes. On the other hand, assuming that most GC synesthetes are capable of this kind of learning, one could reasonably speculate that the main factor limiting its prevalence is simply the availability of consistently colored letters in the environment. Given the demonstrated similarities among all the synesthetes in this sample, that strikes us as a reasonable assumption.

Speculation aside, the question remains, ‘How should the fact that synesthetes can and do learn their letter-color pairs from sources in the environment influence our understanding of synesthesia?’. Our proposal is that GC synesthesia is conditioned mental imagery produced in response to letters [12, 18]. It is akin to the auditory imagery that is evoked by reading for many people [15], though it differs from ordinary imagery of this type in a number of ways, particularly in the automaticity and specificity of the elicited response [12, 19, 20, 21]. Invoking conditioned mental imagery as an explanation for synesthesia does not contradict findings that synesthetic responses can be perceptual, nor data showing that the development of the synesthetic response is dependent on genetic predisposition [22].

Although we propose an explanation of synesthesia that invokes conditioned mental imagery, our intent is not to equate synesthesia with associative learning. Many attempts have been made to induce synesthesia in adults using paired associate training (for example pairing letters with colors) without success [23,24,25]. Such failures do not necessarily mean that associative learning cannot be a mechanism by which synesthetic correspondences are acquired, as our data show. So how can synesthetic pairings be acquired through associative learning, when not all associative learning leads to synesthesia? While this is not yet known, it seems likely that factors about the context of the learning and about the individual are important. The difficulty in inducing synesthesia in adults may reflect differences in age, genetic makeup, and the type of learning and its context. For example, most researchers believe that grapheme-color correspondences come into place as part of learning to read. Developing synesthetes are not trying to learn that ‘A is red’ as participants in artificial training paradigms often are, but rather something like which things are A’s and what do they mean (see Howells for a similar point [26]). It has also been proposed that synesthetes may be ‘stronger’ associative learners, and that this superior learning ability may persist into adulthood [27]).

Learning has also been shown to play a role in other types of synesthesia such as word-taste synesthesia [28], and most types of synesthesia involve culturally learned sequences such as letters, numbers, dates, and pitches. However, there are reasons to be cautious about extrapolating from our result to the claim that synesthetic correspondences in general can be or are often learned from external stimuli. For some types of synesthesia such as pitch-color, synesthetic responses may partially reflect internal correspondences between sensory dimensions (such as pitch and brightness). Similarly, mental number lines may reflect an overlap in the representation of space and number in the brain as has been widely proposed [29]. Learning still matters in both cases, as mastering these sequences appears to be the impetus for the development of synesthesia in some people. However, the particular synesthetic mapping may reflect internal relationships between domains, external contingencies, or idiosyncratic modifications and combinations of these that arise as part of the learning process. Moreover, there is good reason to believe that the various forms of synesthesia may reflect distinct phenomena with different neural and genetic origins. A large-scale study of 18000+ synesthetes (of which some participants here form a subset) suggests there are at least 5 types of synesthetes, with colored sequence synesthesia (colored letters, numbers, weekdays and months) as one subtype [30].

Our findings agree with previous suggestions that GC synesthesia may be better understood in the context of sequence learning [9, 22, 31, 32] and that progress may come from considering the relation between imagery and associative memory [33, 34, 35, 36], rather than randomly ramifying cross-modal correspondences [11]. The data presented here add to a growing body of evidence that the GC synesthetic response is learned, possibly over a fairly long period [32, 37]. Grapheme-color pairings reflect a number of environmental influences, including direct exposure to letter-color pairings, letter frequency, similarities of shape or sound between letters, and language specific semantic factors such as the first letter of color words [6, 7, 8, 10, 38, 39].

Whatever it is that differs between those with grapheme-color synesthesia and those without, the underlying mechanism is clearly sensitive to the external contingencies between the stimulus categories being learned (such as letters) and other possible discriminative stimulus features. While associative learning is not sufficient to explain grapheme-color synesthesia [40], grapheme-color synesthesia reflects a difference in associative learning. Synesthetes not only learn that A sounds like ‘a’, but also that it is red.

## References

[1] Eagleman DM, Kagan AD, Nelson SS, Sagaram D, Sarma SK. A standardized test battery for the study of synesthesia. J Neurosci Methods. 2007;159: 139–145. 16919755 10.1016/j.jneumeth.2006.07.012 PMC4118597

[2] Jewanski J, Simner J, Day SA, Ward J. The development of a scientific understanding of synesthesia from early case studies (1849–73). J Hist Neurosci. 2011;20(4): 284–305. 10.1080/0964704X.2010.528240 22003858

[3] Chabalier. De la pseudochromesthesie. J Medicine de Lyon. 1864;1(2): 92–102.

[4] Calkins MW. A statistical study of pseudo-chromesthesia and of mental forms. Am J Psychol. 1893;5: 439–464.

[5] Galton F. Visualized Numerals. J Anthropol Inst. 1881;10: 85–102.

[6] Rich AN, Bradshaw JL, Mattingley JB. A systematic, large-scale study of synaesthesia: implications for the role of early experience in lexical-color associations. Cognition. 2005;98: 53–84. 16297676 10.1016/j.cognition.2004.11.003

[7] Beeli G, Esslen M, Jancke L. Frequency Correlates in Grapheme-Color Synesthesia. Psychol Sci. 2007;18(9): 788–792. 17760774 10.1111/j.1467-9280.2007.01980.x

[8] Simner J. Non-Random associations of graphemes to colours in synaesthetic and non-synaesthetic populations. Cogn Neuropsychol. 2005;22(8): 1–17. 10.1080/02643290500200122 21038290

[9] Watson MR, Akins KA, Spiker C, Crawford L, Enns JT. Synesthesia and learning: a critical review and novel theory. Front Hum Neurosci. 2014;8(98). 10.3389/fnhum.2014.00098 24592232 PMC3938117

[10] Asano M, Yokosawa K. Grapheme learning and grapheme-color synesthesia: toward a comprehensive model of grapheme-color association. Front Hum Neurosci. 2013;7. 10.3389/fnhum.2013.00757 PMC3822291 24273504

[11] Witthoft N, Winawer J. Synesthetic colors determined by having colored refrigerator magnets in childhood. Cortex. 2006;42: 175–183. 16683491 10.1016/s0010-9452(08)70342-3

[12] Witthoft N, Winawer J. Learning, memory, and synesthesia. Psychol Sci. 2013;24(3): 258–65. 10.1177/0956797612452573 23307940 PMC3648671

[13] Spector F, Maurer D. Synesthesia: A new approach to understanding the development of perception. Dev Psychol. 2009;45(1): 175–89. 10.1037/a0014171 19210000

[14] Barnett KJ, Finucane C, Asher JE, Bargary G, Corvin AP, Newell FN, et al Familial patterns and the origins of individual differences in synaesthesia. Cognition. 2008;106(2): 871–893. 17586484 10.1016/j.cognition.2007.05.003

[15] Alexander JD, Nygaard LC. Reading voices and hearing text: Talker specific auditory imagery in reading. J Exp Psychol: Hum Percept Perform. 2008;34(2): 446–59. 10.1037/0096-1523.34.2.446 18377181

[16] Berlin B, Kay P. Basic color terms, their universality and evolution. Berkeley: University of California Press; 1969 10.3758/s13414-010-0055-9

[17] Barnett KJ, Newell FN. Synaesthesia is associated with enhanced, self-rated visual imagery. Conscious and Cogn. 2008;17: 1032–1039. 17627844 10.1016/j.concog.2007.05.011

[18] Galton F. Mental Imagery. Fortnightly Review. 1880;28: 312–324.

[19] Spence C, Deroy O. Crossmodal mental imagery In: Lacey S, Lawson R, editors. Multisensory Imagery. Springer; 2013 p. 157–183.

[20] Tyler C. Varieties of synesthetic experience In: Robertson L, Sagiv N, editors. Synesthesia: Perspectives from Cognitive Neuroscience. Oxford: Oxford University Press; 2005 p. 34–44.

[21] Martino G, Marks LE. Synesthesia: Strong and weak. Curr Dir Psychol Sci. 2001;10(2): 62–65.

[22] Tomson S, Avidan N, Lee K, Sarma AK, Tushe R, Milewicz D, et al The genetics of colored sequence synesthesia: Suggestive evidence of linkage to 16q and genetic heterogeneity for the condition. Behav Brain Res. 2011;223: 48–52. 10.1016/j.bbr.2011.03.071 21504763 PMC4075137

[23] Rothen N, Meier B. Acquiring synaesthesia: Insights from training studies. Front Hum Neurosci. 2014;8(109). 10.3389/fnhum.2014.00109 24624072 PMC3939620

[24] Colizoli O, Murre JMJ, Rouw R. Pseudo-synesthesia through reading books with colored letters. PLoS One. 2012;7(6). 10.1371/journal.pone.0039799 PMC3384588 22761905

[25] Meier B, Rothen N. Training grapheme-colour associations produces a synaesthetic Stroop effect, but not a conditioned synaesthetic response. Neuropsychologia. 2009;47(4): 1208–1211. 19350712 10.1016/j.neuropsychologia.2009.01.009

[26] Howells TH. The experimental development of color-tone synesthesia. J of Exp Psychol. 1944;34(2): 87–103.

[27] Yon D, Press C. Back to the future: Synaesthesia could be due to associative learning. Front in Psychol. 2014;5(72). 10.3389/fpsyg.2014.00072 25071668 PMC4083340

[28] Ward J, Simner J. Lexical-gustatory synaesthesia: Linguistic and conceptual factors. Cognition. 2003;89(3): 237–261. 12963263 10.1016/s0010-0277(03)00122-7

[29] Cohen-Kadosh R, Henik A. Can synaesthesia research inform cognitive science? Trends Cogn Sci. 2007;11(4): 177–184. 17331789 10.1016/j.tics.2007.01.003

[30] Novich S, Cheng S, Eagleman DM. Is synaesthesia one condition or many? A large-scale analysis reveals subgroups. J Neuropsychol. 2011;5: 353–371. 10.1111/j.1748-6653.2011.02015.x 21923794

[31] Pariyadath V, Plitt MH, Churchill SJ, Eagleman DM. Why overlearned sequences are special: Distinct neural networks for ordinal sequences. Front Hum Neurosci. 2012;6(328). 10.3389/fnhum.2012.00328 23267320 PMC3526771

[32] Simner J, Harrold J, Creed H, Monro L, Foulkes L. Early detection of markers for synaesthesia in childhood populations. Brain. 2009;132: 57–64. 10.1093/brain/awn292 19015159

[33] Leuba C. Images as conditioned sensations. J Exp Psychol. 1940;26(3): 345–351.

[34] Paivio A. Mental imagery in associative learning and memory. Psychol Rev. 1969;76(3): 241–263.

[35] Bower GH. Mental imagery and associative learning In: Gregg L, editor. Cognition in Learning and Memory. New York:John Wiley & Sons; 1972 p. 51–88.

[36] Wheeler ME, Petersen SE, Buckner RL. Memory’s echo: Vivid remembering reactivates sensory-specific cortex. Proc Natl Acad Sci U S A. 2000;97(20): 11125–11129. 11005879 10.1073/pnas.97.20.11125 PMC27159

[37] Simner J, Baine A. A longitudinal study of grapheme-color synesthesia in childhood: 6/7 years to 10/11 years. Front Hum Neurosci. 2013;7(603). 10.3389/fnhum.2013.00603 24312035 PMC3826064

[38] Brang D, Romke R, Ramachandran VS, Coulson S. Similarly shaped letters evoke similar colors in grapheme-color synesthesia. Neuropsychologia. 2011;49: 1355–1358. 10.1016/j.neuropsychologia.2011.01.002 21219918

[39] Mroczko-wasowicz A, Nikolic D. Colored alphabets in bilingual synesthetes In: Simner J, Hubbard E, editors. Oxford Handbook of Synaesthesia. Oxford:Oxford University Press; 2013 p.165–180.

[40] Marks LE, Odgaard EC. Developmental constraints on theories of synesthesia In: Robertson L, Sagiv N, editors. Synesthesia: Perspectives from Cognitive Neuroscience. Oxford: Oxford University Press; 2005 p. 214–236.

